# Controlling social desirability bias: An experimental investigation of the extended crosswise model

**DOI:** 10.1371/journal.pone.0243384

**Published:** 2020-12-07

**Authors:** Julia Meisters, Adrian Hoffmann, Jochen Musch

**Affiliations:** Department of Experimental Psychology, University of Duesseldorf, Duesseldorf, Germany; Ghent University, BELGIUM

## Abstract

Indirect questioning techniques such as the crosswise model aim to control for socially desirable responding in surveys on sensitive personal attributes. Recently, the extended crosswise model has been proposed as an improvement over the original crosswise model. It offers all of the advantages of the original crosswise model while also enabling the detection of systematic response biases. We applied the extended crosswise model to a new sensitive attribute, campus islamophobia, and present the first experimental investigation including an extended crosswise model, and a direct questioning control condition, respectively. In a paper-pencil questionnaire, we surveyed 1,361 German university students using either a direct question or the extended crosswise model. We found that the extended crosswise model provided a good model fit, indicating no systematic response bias and allowing for a pooling of the data of both groups of the extended crosswise model. Moreover, the extended crosswise model yielded significantly higher estimates of campus Islamophobia than a direct question. This result could either indicate that the extended crosswise model was successful in controlling for social desirability, or that response biases such as false positives or careless responding have inflated the estimate, which cannot be decided on the basis of the available data. Our findings highlight the importance of detecting response biases in surveys implementing indirect questioning techniques.

## Introduction

Surveys of sensitive personal attributes often rely on self-reports. However, socially desirable responding, that is, the tendency to answer in accordance with social norms rather than truthfully, may result in underestimates of the prevalence of socially undesirable attributes and overestimates of the prevalence of socially desirable attributes [1, 2]. To address this problem, indirect questioning techniques such as the randomized response technique (RRT [3]) have been proposed. Based on an experimental randomization procedure, the RRT provides prevalence estimates of sensitive attributes on the sample level while preserving the confidentiality of individual responses. A comprehensive meta-analysis [4] confirmed the usefulness of this approach and concluded that the RRT provides more valid prevalence estimates than direct questioning (DQ). However, in some studies, the RRT did not work as intended, and provided prevalence estimates that did not differ significantly from DQ estimates, were lower than DQ estimates, or even negative and thus outside of the admissible range [5–9]. As a consequence of the mixed results with regard to the validity of the RRT, advanced techniques such as the crosswise model (CWM [10]) have been proposed that aim at improving comprehensibility and the perceived protection of the respondents’ privacy.

The CWM presents respondents with a statement regarding a sensitive behavior or attitude (e.g., “Many Muslim students behave in misogynist ways”) in order to estimate its prevalence π, and a non-sensitive statement with known prevalence *p* that is used for randomization (e.g., “I was born in November or December”). To preserve confidentiality, respondents are not asked to agree or disagree with either of these statements individually, but to choose one of the following two combined answer options: “I agree with *both* of the statements or *none* of the statements” versus “I agree with *exactly one* of the statements (irrespective of which one)”. Compared to other indirect questioning techniques, such as the Unmatched Count Technique [11], the Stochastic Lie Detector [12] and the Cheating Detection Model [13], the CWM is easier to comprehend [14]. Moreover, the CWM is easier to provide instructions for, since in contrast to many RRTs it does not require an external randomization device [10]. The CWM has led to significantly higher and thus—according to the “more is better” criterion—presumably more valid prevalence estimates than direct questions in a number of studies investigating sensitive attributes such as xenophobia [15, 16], plagiarism [17], tax evasion [18, 19], the use of anabolic steroids by bodybuilders [20], the intention to vote for the far-right German party Alternative for Germany [21], distrust in the Trust Game [22] and prejudice against female leaders [23]. Moreover, the CWM was able to accurately estimate the known prevalence of experimentally induced cheating behavior [24]. Participants were asked to indicate the number of anagrams they had been able to solve within a defined time frame. However, in a pretest and unknown to the participants, the last of the three anagrams had proven to be virtually unsolvable (with a probability of finding the solution of <1%). Participants nevertheless indicating that they had solved all three anagrams could therefore be categorized as cheaters, and the prevalence of cheating could be determined on sample level. Subsequently, participants were questioned about their cheating behavior in either CWM or DQ format. While the CWM question successfully recovered the known prevalence of cheating, the DQ format provided a significant underestimate.

These positive evaluations of the CWM might be partly attributable to the model’s response symmetry. Response symmetry means that both answers can stem from carriers as well as noncarriers of the sensitive attribute. In contrast to asymmetric models such as the Triangular model (TRM [10]) or the forced-choice RRT [25, 26], symmetric models such as the CWM encourage honest responding because no ‘safe’ answer option is available that excludes the possibility of a respondent being a carrier of the sensitive attribute. Recently, however, the validity of the CWM has been questioned because false positives have been observed in CWM surveys [27–29]. Like direct self-reports and other indirect questioning techniques, the CWM is based on the assumption that respondents will follow the model’s instructions. However, this assumption may be violated under certain conditions [13, 30, 31]. A potential reason for instruction non-adherence is that some respondents might not understand or trust indirect questioning procedures [14, 32]. Moreover, a specific kind of instruction non-adherence in the CWM is a systematic preference for one of the two answer options (“I agree with *both* of the statements or *none* of the statements” versus “I agree with *exactly one* of the statements (irrespective of which one)”). Such a preference is likely to occur when, for example, respondents subjectively perceive one answer option less incriminating than the other [33]; in the original CWM, it can however not be detected. Two approaches are available to address the problems of false positives and instruction non-adherence. First, at least in higher-educated samples, it might be possible to reduce false positives by offering detailed instructions and implementing comprehension checks to ensure that all instructions are properly understood [29]. Alternatively, the extended CWM (ECWM [33]) can be applied to test for systematic response biases.

**Fig 1:**
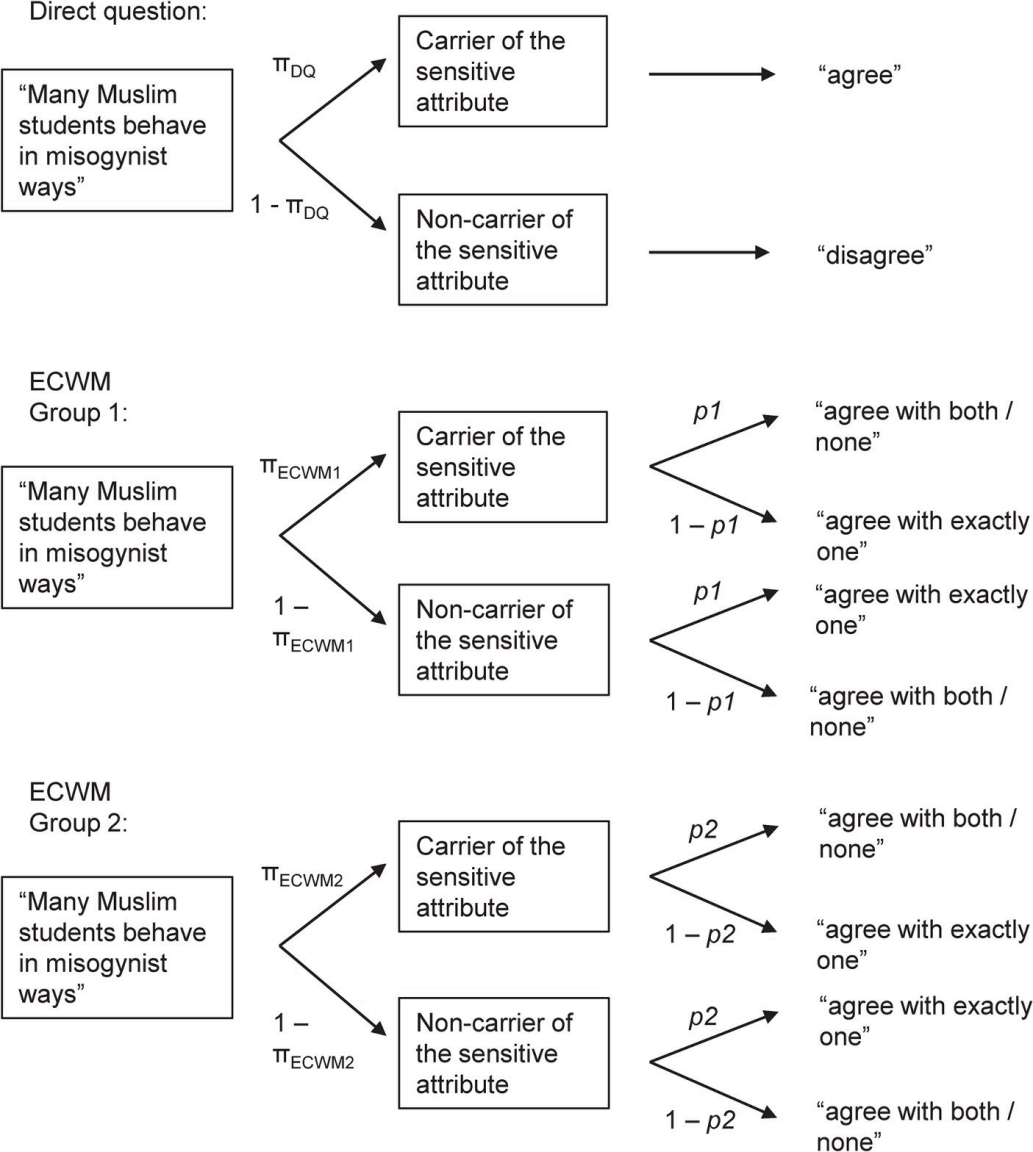
Tree diagrams for direct questioning and the Extended Crosswise Model (ECWM). The parameter π represents the unknown prevalence of the sensitive attribute and the parameter *p1* and *p2* represent the known randomization probabilities. In the current study, randomization probabilities as obtained from official birth statistics provided by the German Federal Statistical Office [34] were *p1* = .158, and *p2* = .842, respectively.

The ECWM offers all the advantages of the CWM and has identical statistical efficiency in parameter estimation, but additionally enables the identification of systematic preferences for one of the two answer options. The central idea is to apply the CWM to two non-overlapping groups with reversed randomization probabilities *p1* and *p2* (see Fig 1). Since the sensitive statement is identical for both groups to which the respondents are randomly assigned, the prevalence π of the sensitive attribute should not differ between groups (π_ECWM_1_ = π_ECWM_2_). Because two independent answer frequencies can be observed in the two groups, the resulting model has one degree of freedom and its fit can therefore be tested. If the prevalence estimates π_ECWM_1_ and π_ECWM_2_ do not significantly differ from one another, they can be pooled and are readily interpretable [33]. If a model misfit is indicated by significant differences in the prevalence estimates π_ECWM_1_ and π_ECWM_2_, the prevalence estimates should not be interpreted because the misfit indicates that a substantial share of respondents did not adhere to the instructions and exhibited a systematic preference for one of the two answer options [33]. The ECWM even allows for detecting systematic preferences for one of the two answer options if this preference occurs only among carriers or among non-carriers of the sensitive attribute. So far, the ECWM has only been applied by its original authors [33]. In their study, two sensitive questions were used: One question pertained to the use of performance-enhancing substances. This question was asked under optimal conditions; consequently, the ECWM fitted the data well, and the estimate could be pooled across the two ECWM groups. A second question asked about whether participants had been infected with a sexually transmitted disease. This question was asked under conditions that increased the likelihood of instruction non-adherence (a non-optimal randomization scheme); as expected, the ECWM did not fit the data for this question well, indicating a violation of the underlying model assumptions, and the inadmissibility of determining a pooled estimate. However, prevalence estimates of the ECWM have never been compared with prevalence estimates of a direct questioning control condition. We therefore conducted a conceptual replication of the findings by Heck et al. [33] using a large sample and adding an important direct question control group that was missing in the original article and that allowed the first experimental comparison between ECWM and DQ prevalence estimates. Using the model test made possible by the ECWM, we were able to show that there was no systematic response bias towards one of the available answer options in the present sample. We could thus show that the higher estimates in the ECWM condition are either valid, or that they were produced by another bias than the systematic bias towards one of the available answer options that can be detected by using the ECWM. In doing so, we made use of the fact that the major advantage of the ECWM—the possibility of conducting a model test—comes at the small price of only a minor inconvenience, namely that two questionnaires with different non-sensitive statements have to be prepared instead of one. Finally, our study also extends the previous findings by Heck et al. [33] to a new sensitive attribute, campus islamophobia, thus broadening the empirical base on which the ECWM can be evaluated.

### Assessing the prevalence of Islamophobia

Islamophobia is defined as a negative attitude towards, or fear of, Islam as a religion and people of Muslim faith. Muslims currently experience high levels of prejudice and discrimination in Western societies due to their religious affiliation. Surveys in several European countries show that attitudes towards Muslims are far more negative than attitudes towards members of other religions [35–38]. In Germany, people of Muslim faith are often stereotyped as a problematic, delinquent and aggressive minority [37–39]. Consequently, attitudes towards Muslims in Germany are even more negative than in other European countries [40], and fears of Islamist terrorism are widespread [36, 37]. A common concern is that Islam promotes intolerance and is therefore incompatible with Western open societies [36, 37], particularly with respect to gender equality [38]. In representative samples of eight European countries, 72%-82% of respondents agreed with the statement “The Muslim views on women are contrary to our values” [38] and in two representative German samples, more than 80% of respondents believed that Islam is characterized by discrimination against women [37–39].

In recent years, blatantly negative attitudes towards foreigners or Muslims seem to have been increasingly replaced by subtler forms of prejudice [41–43]. This could possibly be due to a genuine change in attitudes on a broad, societal level. Alternatively, prejudiced individuals may become increasingly aware of the growing social undesirability of their views and may therefore be more reluctant to express more blatant forms of prejudice openly [44]. Following this reasoning, estimates for the prevalence of Islamophobia based on direct self-reports are likely underestimates of the true value, and indirect questioning techniques may help to obtain more valid estimates [15, 16, 31, 45]. If the ECWM is suitable as a new means of controlling for social desirability, it should provide higher and thus potentially more valid estimates for the prevalence of Islamophobia than a conventional direct question.

The results of numerous studies indicate that explicit prejudice against Muslims and other minorities is less common among better educated [31, 46–48] and younger respondents [41, 49]. In a study by Stocké [44], younger and better educated respondents also reported higher perceived pressure to answer in accordance with social norms. However, even when controlling for social desirability, prejudice seems to be less prevalent among higher-educated compared to lower-educated respondents [31, 44, 46]. Nevertheless, in a study by Kassis, Schallié, Strube, and van der Heyde [50], more than 20% of respondents from a German university sample expressed strong anti-Muslim attitudes. Thus, even though higher-educated respondents seem to exhibit less prejudice towards Muslims or other minorities, such prejudice is still common; moreover, socially desirable responding is demonstrably an issue, especially among higher-educated respondents. This made Islamophobia a particularly well-suited subject for our experimental investigation of the ECWM in a German university sample.

## Methods

### Participants

The initial sample consisted of 1,629 students from the University of Duesseldorf, Germany. Due to item nonresponse, 98 respondents (6.02% of the initial sample) had to be excluded from further analyses. Dropout rates were significantly higher in the DQ (7.65%) than in the ECWM (5.19%) condition, although this effect was rather small, χ²(1) = 3.91, *p* = .048, *Cramer’s V* = .05. The responses of 181 Muslims who participated in our study were excluded because we wanted to investigate Islamophobia among non-Muslim respondents.

The final sample consisted of *N* = 1,361 respondents (55.69% female). Age was only assessed in broad categories to increase the confidentiality of responses, and was distributed as follows: younger than 20 years (55.55%), 20–29 years (40.71%), 30–39 years (1.91%), 40–49 years (0.66%), 50–59 years (0.37%) and 60 and above (0.81%). Twice as many respondents were assigned to the ECWM condition (*n* = 911; 66.94%) as to the DQ condition (*n* = 450; 33.06%) to compensate for the lower efficiency of indirect questioning techniques [51]. This lower efficiency is a result of the randomization procedure which introduces additional variance to the estimates, thereby inflating their standard error [12, 51]. Within the ECWM group, we assigned *n* = 455 respondents to the ECWM condition with randomization probability *p1* = .158 and *n* = 456 to the ECWM condition with randomization probability *p2* = .842. Randomization probabilities *p1* and *p2* were obtained from official birth statistics provided by the German Federal Statistical Office [34]. Respondents in the two ECWM conditions (*p1* and *p2*) did not differ with regard to age group, χ²(5) = 6.38, *p* = .271, *Cramer’s V* = .08, or gender, χ²(1) = 0.80, *p* = .370, *Cramer’s V* = .03. Comparisons between the two questioning technique groups (DQ vs. ECWM) did also not indicate significant differences with regard to age group, χ²(5) = 3.78, *p* = .581, *Cramer’s V* = .05, or gender, χ²(1) = 0.15, *p* = .695, *Cramer’s V* = .01.

### Survey design

Between lectures, respondents filled in a one-page questionnaire consisting of the experimental question and additional questions about their gender, age group, and religious affiliation (Muslim vs. non-Muslim). The experimental question on the presumably sensitive topic of campus Islamophobia was presented in either the DQ format or the ECWM format. The survey also included three additional questions pertaining to the participant’s political orientation, their frequency of contact with Muslims, and their perception of Muslims’ attitudes towards gender roles. However, these questions did not moderate any of our main findings and are therefore not discussed further. Respondents were unaware of the experimental design of the study, and did not know that other respondents were presented with a different question format. Respondents were asked to work in silence and not to talk to their neighbors when filling out the survey. Allocation of the respondents to the experimental conditions was performed by printing and sorting all questionnaire versions in alternating order (DQ, ECWM *p1*, ECWM *p2*) prior to distributing them to the students in the lecture halls. This pseudo-randomization made sure that neighboring students received different versions of the questionnaire and that conditions were not confounded with seating place or other variables. The non-significant differences with regard to the demographic variables of the participants in different experimental groups confirmed that the desired random distribution of participants across experimental conditions was indeed achieved. The survey was carried out in accordance with the revised Declaration of Helsinki [52] and the ethical guidelines of the German Society for Psychology [53]. In Germany, there is no binding obligation that research projects can only be carried out after approval by an ethics committee. Participation in the present study could not have any negative consequences for the respondents, and anonymity was ensured at all times. The respondents participated voluntarily and after informed consent was obtained. There was no risk that participation could cause any physical or mental damage or discomfort to participants beyond their normal everyday experiences. Therefore, ethics committee approval was not required according to the “Ethical Research Principles and Test Methods in the Social and Economic Sciences” formulated by the Ethics Research Working Group of the German Data Forum [54] and the “Ethical Recommendations of the German Psychological Society” [55]. Prior to their participation, all respondents were informed of the strict anonymization of all data, and consented to participate on a voluntary basis without receiving financial compensation.

A priori power considerations based on Ulrich et al. [51] indicated that a four-digit sample size would ensure sufficient statistical power (1-ß ≥ .80) for the required prevalence estimates and parameter comparisons. Post-hoc power analyses based on the main effect observed for questioning technique (prevalence estimate in ECWM condition: 21.19%; prevalence estimate in DQ condition: 10.89%) confirmed that our sample size was sufficient to achieve high statistical power (1-ß = .97).

### Sensitive question formats

#### DQ

Respondents in the DQ condition were simply presented with the sensitive statement (“Many Muslim students behave in misogynist ways”) and had to indicate whether they agreed with this statement or not.

#### ECWM

In each of the ECWM conditions, the sensitive statement was paired with one of two non-sensitive statements. In the group with randomization probability *p1 =* .158, the non-sensitive statement read: “My father was born in November or December”; and in the group with randomization probability *p2* = .842, it read: “My father was born between January and October” (*p1* and *p2* were obtained from official birth statistics provided by the German Federal Statistical Office [34]). Respondents were asked to indicate whether they agreed with “*both* of the statements or *none* of them”, or whether they agreed with “*exactly one* statement (irrespective of which one)”.

### Statistical analyses

To obtain and compare parameter estimates, we established multinomial processing trees [56, 57] for both questioning techniques, as detailed in, for example [12, 58, 59]. A graphical representation of the processing trees for the DQ and ECWM conditions is shown in Fig 1. Based on the empirically observed answer frequencies, parameter estimates were obtained using the expectation maximization algorithm [60, 61] as implemented in the software multiTree [62]. To compare the parameter estimates, an unrestricted baseline model was compared to a restricted alternative model in which the respective parameters were set to be equal (e.g. π_ECWM_ = π_DQ_) or set to a certain constant (e.g., 0). Model fit was assessed via the asymptotically χ²-distributed log-likelihood ratio *G*². Significant differences in model fit indicated that the imposed restriction was inadmissible and that the respective parameters differed significantly from each other (π_ECWM_ ≠ π_DQ_).

## Results

The ECWM fit the empirically observed data well, *G*²(1) = 0.10, *p* = .756, indicating that the prevalence estimates did not differ between both groups of the ECWM (group with randomization probability *p1*: 21.89%, *SE* = 3.16%; group with randomization probability *p2*: 20.50%, *SE* = 3.13%). These results illustrate that respondents did not show any systematic bias towards one of the available answer options and that a simple one-group CWM design with either *p1* or *p2* would have resulted in very similar estimates. Pooling across the two groups with different randomization probabilities resulted in a prevalence estimate of 21.19% (*SE* = 2.23%). The prevalence estimate of campus Islamophobia was significantly higher in the ECWM (21.19%; *SE* = 2.23%) than in the DQ (10.89%; *SE* = 1.47%) condition, Δ*G*²(1) = 14.69, *p* < .001, and both estimates were significantly higher than zero, DQ: Δ*G*²(1) = 1495.46, *p* < .001; ECWM: Δ*G*²(1) = 119.40, *p* < .001.

## Discussion

We report the first conceptual replication of the findings by Heck et al. [33] using a large sample and adding the direct question control group that allowed the first experimental comparison between ECWM and DQ prevalence estimates. Heck et al. [33] applied the ECWM to estimate the prevalence of the use of performance-enhancing drugs and having been infected with a sexually transmitted disease. They found that the ECWM provided an adequate fit to the data for a question regarding performance-enhancing drugs, but not for a question regarding the sexually transmitted disease. The current study extends the findings by Heck et al. [33] with further experimental evidence regarding the performance of the ECWM in the context of campus Islamophobia. A good model fit indicated that the ECWM prevalence estimates were not distorted by any systematic bias in favor of one of the two answer options.

Although in the present study, we did not observe a significant difference between the prevalence estimates of both ECWM groups, this problem could easily occur in other studies, as for example for one sensitive question in the study reported by Heck et al. [33]. The absence of a systematic response bias is an important prerequisite for obtaining valid prevalence estimates; however, when applying the standard CWM, whether this precondition is actually met cannot be tested. In contrast, the ECWM offers the advantage of allowing the detection of problematic response biases without negatively effecting the model’s efficiency. If a response bias is detected, this raises an important flag that the prevalence estimates cannot be trusted. If, however, no response bias is detected (as was the case in the current study), a single estimate pooled across groups can be obtained. This major advantage of the ECWM comes at the small price of a minor inconvenience, namely that in paper-pencil applications, two questionnaires with different non-sensitive statements representing the randomization probabilities *p1* and *p2* have to be prepared.

In contrast to a direct question, the ECWM yielded higher prevalence estimates of campus Islamophobia. According to the “more-is-better”-criterion, which is based on the assumption that higher prevalence estimates for socially undesirable attributes are more valid and less distorted by social desirability bias [4], these higher ECWM estimates could be interpreted as more valid. Under this assumption, our results suggest that Islamophobia was perceived as a sensitive topic, and that respondents’ willingness to honestly admit to Islamophobic attitudes was lower in the direct questioning compared to the ECWM condition. Following this rationale, previous studies investigating Islamophobia based on direct self-reports might have provided underestimates of the true prevalence, as the results in these studies were potentially biased by socially desirable responding [25–29, 40]. However, the evidence obtained by applying the „more-is-better“-criterion is limited, since only one possible source of bias can be controlled by using the ECWM and alternative explanations for higher prevalence estimates cannot be ruled out, as for example false positives or random responding.

The CWM has recently been observed to produce false positives under certain conditions [27–29]; non-carriers of a sensitive attribute were wrongly categorized as carriers. Moreover, the CWM has also been shown to sometimes produce false negatives by wrongly categorizing some carriers of a sensitive attribute as non-carriers [29]. Since the present study applied only a weak (“more-is-better”) and not a strong validation criterion in which the individual status with regard to the sensitive characteristic was known for each respondent, it is impossible to tell whether false positives or false negatives may have influenced the current results. We therefore recommend that future studies seek to create conditions under which a strong validation can be conducted. To this end, researchers could experimentally induce a sensitive attribute with known prevalence to obtain an external criterion [24]. However, it is important to note that so-called “strong” validation studies in which the true prevalence of a sensitive attribute is presumably known may also have methodological shortcomings, and comparing the results of alternative and complementary approaches might be helpful to arrive at a realistic and comprehensive assessment of the validity of indirect questioning techniques.

While some strong validation studies have indeed demonstrated a tendency of indirect questioning techniques such as the CWM to produce false positives [27, 28], other strong validations found that the model accurately recovered the known prevalence of sensitive [24] and non-sensitive attributes [15]. These findings contradict the assumption that models such as the CWM have a general tendency to overestimate the prevalence of a given attribute. Rather, they reveal that the variables responsible for the differences between these strong validations are not yet understood and have to be examined in future research before final conclusions regarding their (in-)validity can be drawn. In a similar vein, future studies still have to identify the conditions under which weak validation studies do provide valid or invalid estimates.

False positives or false negatives might at least partly be explained by careless responding, which is likely to occur when respondents do not understand what they have to do. If a substantial proportion of respondents answered randomly, CWM estimates would be biased towards 50%, irrespective of the true prevalence of the sensitive attribute [63]. Both direct and indirect evidence for careless responding in CWM conditions has been reported in recent studies [29, 63, 64]. Some studies showed that the CWM yielded lower prevalence estimates for a socially desirable attribute than a direct question, or prevalence estimates significantly above zero for attributes with a known prevalence of zero [27, 64, 65]. These studies indicate that careless responding might introduce a potential bias threatening the validity of (E)CWM estimates. This bias is however different from a systematic preference for one of the two answer options and can therefore not be detected by the ECWM [33].

In the present study, the order in which options were presented was fixed; therefore, option order was confounded with the position of the answer. Theoretically, the absence of a significant difference between the two arms might therefore have been the result of opposing effects of order and question type that cancelled each other out. However, this explanation would require that both effects happened to be about equal in size, but in opposite directions. We consider this possibility highly unlikely given that in a recent study, Höglinger and Diekmann [27] did not find any evidence for an order effect. Moreover, any remaining systematic answer preference for one of the answer options would have been detected by the ECWM, which was not the case in the current study.

Another limitation might be related to the non-sensitive attribute used in the present study. Knowledge or memory of their father’s month of birth might be less than perfect for some of the respondents; however, such cases are presumably rare. We decided to ask respondents about the month of birth of their father rather than about their own month of birth because the latter would potentially have interfered with the assured protection of their privacy.

In Germany, two recent studies found markedly higher shares of respondents directly admitting to being prejudiced against Muslims than the present study. In representative German samples, the share of respondents agreeing to the statement “The Muslim opinion on women contradicts our values” was 76.1% [38], and more than 80% of respondents associated Islam with discrimination against women [37, 39]. The relatively low rate of Islamophobic responses in our study may have been caused by three factors. First, we used a different question wording and thus a different operationalization of the sensitive statement. Second, we explicitly asked about prejudice against “Muslim university students”, and thus a higher-educated and more progressive subgroup of Muslims against whom prejudice might be less prevalent. Third, unlike previous studies, we employed a student sample comprised of younger and more highly-educated respondents. This difference in samples might explain our relatively low prevalence estimates because several studies suggest that higher-educated samples are generally less prejudiced [31, 46, 48]. However, the ECWM results indicate that prejudice against Muslims was still prevalent in more than 20% of our highly-educated university sample.

## Conclusion

In a conceptual replication of the study by Heck et al. [33], we applied the recently proposed extended crosswise model (ECWM) to assess the prevalence of campus islamophobia. An assessment of model fit indicated that the respondents showed no systematic response bias towards one of the response alternatives. Although in the present study, we did not observe a significant difference between the prevalence estimates obtained in the two ECWM groups, this problem could well occur in other studies. In contrast to the original CWM, the ECWM offers the advantage of being able to detect such problematic systematic biases, while having no disadvantage with regard to efficiency and taking only slightly more effort to prepare an additional questionnaire. This is however only a minor inconvenience considering that unlike the CWM, the ECWM allows for detecting systematic response biases. The ECWM led to significantly higher prevalence estimates of campus Islamophobia than a conventional direct question. According to the “more-is-better”-criterion, this would have to be interpreted as a hint that direct self-reports of Islamophobia might be distorted by socially desirable responding and that indirect questioning techniques such as the ECWM can help to control for socially desirable responding. However, since the present study only applied a weak validation criterion, we cannot rule out alternative explanations such as careless responding for the seemingly positive evaluation of the ECWM. The validity of the ECWM should therefore be assessed further in strong validation studies that compare prevalence estimates with the known prevalence of a sensitive attribute. Moreover, improved methods to detect random answer behavior should be developed to better inform the evaluation of the validity of randomized-response models that might be affected by such behavior. The ECWM seems to be a promising candidate for these further validation studies, because it is the only model that allows for the detection of systematic response biases without a loss of statistical efficiency.

## Supporting information

S1 File MultiTree equations for the estimation of π in a multinomial model. (PDF)

S1 Data Empirically observed answer frequencies used for parameter estimation in multiTree. (PDF)
