# Supplementary material for: Controlling social desirability bias: An experimental investigation of the extended crosswise model
Source: PLoS One. 2020 Dec 7;15(12):e0243384. doi: 10.1371/journal.pone.0243384 (PMC7721152; doi:10.1371/journal.pone.0243384)
Supplement: S1 File — (PDF) [file pone.0243384.s001.pdf]

MultiTree equations

MultiTree equations for the estimation of π (Pi) in a multinomial model. Parameter p1 denotes the known probability of being born in November or December (p1 = .158) and parameter p2 denotes the known probability of being born between January and October (p2 = .842, Pötzsch, 2012). ECWM = extended crosswise model, DQ = direct questioning.

```
ECWM_p1      ECWM_p1_bothnone       Pi_ECWM*p1
ECWM_p1      ECWM_p1_one            Pi_ECWM*(1-p1)
ECWM_p1      ECWM_p1_one            (1-Pi_ECWM)*p1
ECWM_p1      ECWM_p1_bothnone       (1-Pi_ECWM)*(1-p1)
ECWM_p2      ECWM_p2_bothnone       Pi_ECWM*p2
ECWM_p2      ECWM_p2_one            Pi_ECWM*(1-p2)
ECWM_p2      ECWM_p2_one            (1-Pi_ECWM)*p2
ECWM_p2      ECWM_p2_bothnone       (1-Pi_ECWM)*(1-p2)
DQ           DQ_agree               Pi_DQ
DQ           DQ_disagree            (1-Pi_DQ)
```
