# Supplementary material for: Controlling social desirability bias: An experimental investigation of the extended crosswise model
Source: PLoS One. 2020 Dec 7;15(12):e0243384. doi: 10.1371/journal.pone.0243384 (PMC7721152; doi:10.1371/journal.pone.0243384)
Supplement: S1 Data — (PDF) [file pone.0243384.s002.pdf]

Data

Empirically observed answer frequencies used for parameter estimation in multiTree (Moshagen, 2010). ECWM = extended crosswise model, DQ = direct questioning.

```
ECWM_p1_bothnone     315
ECWM_p1_one          140
ECWM_p2_bothnone     136
ECWM_p2_one          320
DQ_agree             49
DQ_disagree         401
```
